# Neutralization of SARS-CoV-2 Variants of Concern Harboring Q677H

**DOI:** 10.1128/mBio.02510-21

**Published:** 2021-10-05

**Authors:** Cong Zeng, John P. Evans, Julia N. Faraone, Panke Qu, Yi-Min Zheng, Linda Saif, Eugene M. Oltz, Gerard Lozanski, Richard J. Gumina, Shan-Lu Liu

**Affiliations:** a Center for Retrovirus Research, The Ohio State University, Columbus, Ohio, USA; b Department of Veterinary Biosciences, The Ohio State University, Columbus, Ohio, USA; c Molecular, Cellular and Developmental Biology Program, The Ohio State University, Columbus, Ohio, USA; d Center for Food Animal Health, Animal Sciences Department, OARDC, College of Food, Agricultural and Environmental Sciences and Veterinary Preventive Medicine Department, College of Veterinary Medicine, The Ohio State University, Wooster, Ohio, USA; e Department of Microbial Infection and Immunity, The Ohio State University, Columbus, Ohio, USA; f Department of Pathology, The Ohio State University, Columbus, Ohio, USA; g Division of Cardiovascular Medicine, Department of Internal Medicine, Davis Heart and Lung Research Institute, The Ohio State University, Columbus, Ohio, USA; h Viruses and Emerging Pathogens Program, Infectious Diseases Institute, The Ohio State University, Columbus, Ohio, USA; Columbia University/HHMI

**Keywords:** Q677H, SARS-CoV-2, spike, neutralization, variant of concern

## Abstract

The sensitivity of SARS-CoV-2 variants of concern (VOCs) to neutralizing antibodies has largely been studied in the context of key receptor binding domain (RBD) mutations, including E484K and N501Y. Little is known about the epistatic effects of combined SARS-CoV-2 spike mutations. We now investigate the neutralization sensitivity of variants containing the non-RBD mutation Q677H, including B.1.525 (Nigerian isolate) and Bluebird (U.S. isolate) variants. The effect on neutralization of Q677H was determined in the context of the RBD mutations and in the background of major VOCs, including B.1.1.7 (United Kingdom, Alpha), B.1.351 (South Africa, Beta), and P1-501Y-V3 (Brazil, Gamma). We demonstrate that the Q677H mutation increases viral infectivity and syncytium formation, as well as enhancing resistance to neutralization for VOCs, including B.1.1.7 and P1-501Y-V3. Our work highlights the importance of epistatic interactions between SARS-CoV-2 spike mutations and the continued need to monitor Q677H-bearing VOCs.

## OBSERVATION

Severe acute respiratory syndrome coronavirus 2 (SARS-CoV-2) continues to evolve into new variants of concern (VOCs) with increased transmissibility, pathogenesis, and vaccine resistance (1). Such mutations can have drastic effects on viral spread, as illustrated by the D614G mutation, which emerged early in the pandemic and is now present in nearly all circulating SARS-CoV-2 strains (2, 3). As global vaccination efforts are under way, monitoring the immune escape of VOCs remains a critical priority. Neutralizing antibodies, or antibodies that directly block virus entry (4), are a key measure of protection against SARS-CoV-2 (5–7). Given that neutralizing antibodies often target the receptor binding domain (RBD) (8), recent studies on neutralizing antibody escape by VOCs, including the rapidly spreading B.1.1.7 (United Kingdom, Alpha), B.1.351 (South Africa, Beta), B.1.429 (United States), and P1 (P1-501Y-V3, Brazil, Gamma) variants (9–13), have focused on mutations in the RBD, including E484K, N501Y, and L452R, which have been shown to decrease the efficacy of mRNA vaccines to VOCs (14, 15).

**FIG 1:**
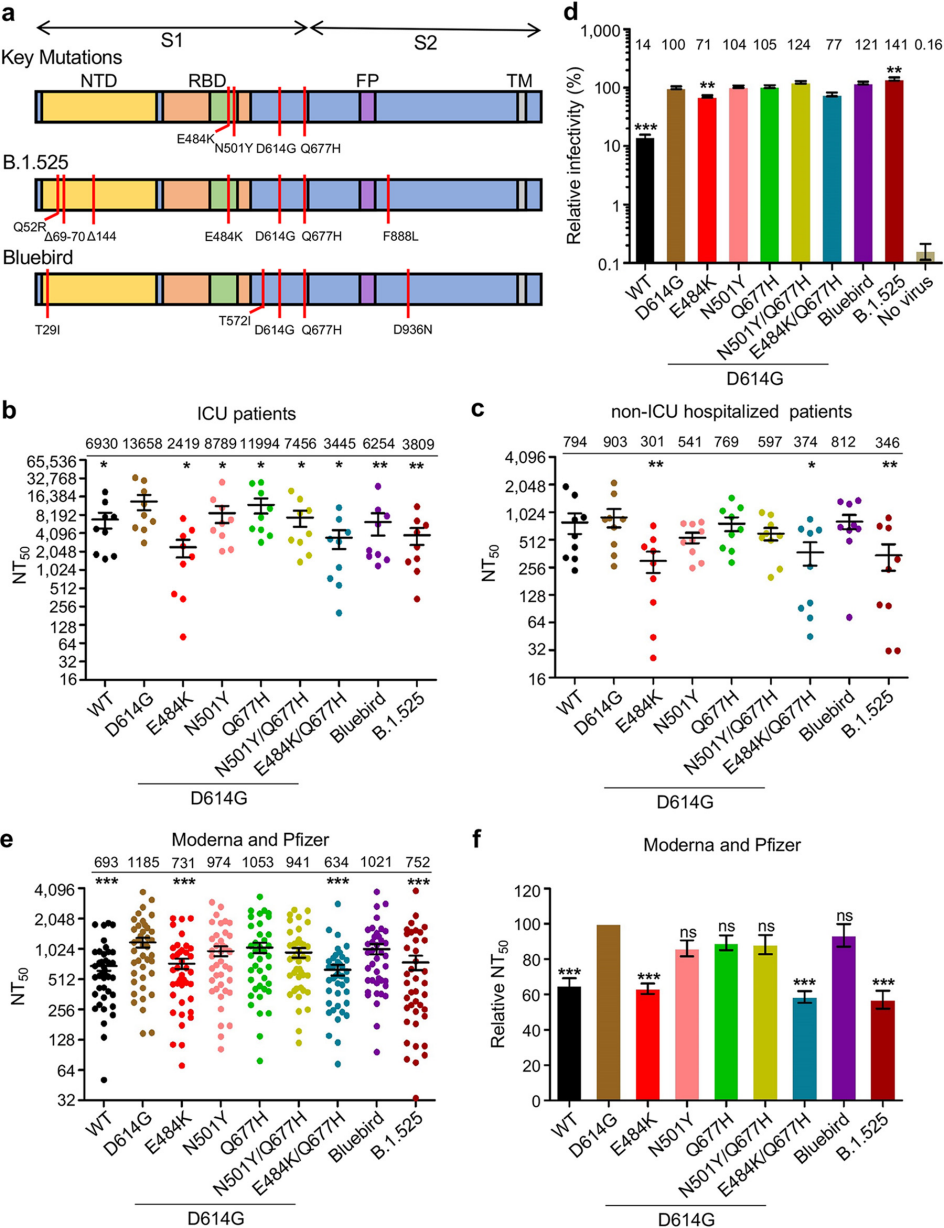
Neutralization of Q677H-bearing SARS-CoV-2 spike-pseudotyped lentivirus by convalescent-phase and vaccinee sera. (a) A schematic representation of the SARS-CoV-2 spike protein is presented that outlines the N-terminal domain (NTD), the receptor binding domain (RBD) with the receptor binding motif in green, the fusion peptide (FP), and transmembrane (TM) regions. Key mutations E484K, N501Y, D614G, and Q677H are indicated. Spike mutations for the B.1.525 and Bluebird variants are shown. (b and c) Luciferase readout from HEK293T-ACE2 cells infected with pseudotyped HIV-1-NL4-3-inGluc virus that had been incubated with serial dilutions of patient serum was used to determine NT_50_ values (also indicated on the top) for sera from 9 COVID-19 ICU patients and 9 non-ICU hospitalized COVID-19 patients against the indicated SARS-CoV-2 spike pseudotyped viruses, including B.1.525 and Bluebird. (d) Infectivity of the lentiviral pseudotypes bearing the indicated spikes of interest on HEK293T-ACE2 cells. Relative infectivity (also indicated on the top) was calculated by setting the value of D614G to 100. (e and f) NT_50_ values and NT_50_ values relative to the D614G virus were determined for 40 vaccinee serum samples collected 3 to 4 weeks after their second dose of Moderna (*n* = 20) or Pfizer (*n* = 20) vaccine with significance being determined by one-way repeated-measures analysis of variance (ANOVA) with Bonferroni posttest. All comparisons were made against D614G; *, *P* < 0.05; **, *P* < 0.01; ***, *P* < 0.001; ns, not significant.

In comparison, emerging B.1.525 (Nigeria) and Bluebird (United States) variants containing Q677H have received less attention, despite maintaining a strong prevalence in some parts of the United States, especially the Midwest and Southeast areas, as well as Nigeria (16–18). Both the B.1.525 and Bluebird variants harbor a key S1 non-RBD mutation, Q677H, with the B.1.525 variant also possessing the E484K RBD mutation (Fig. 1a). Importantly, the Q677H mutation is also present in some isolates of VOCs, including B.1.1.7, B.1.351, and P1 (19). The possible role of Q677H in modulating viral infectivity and SARS-CoV-2 sensitivity to antibody neutralization is currently unknown, a particular concern if it emerges in an existing VOC. Here, we examine the infectivity and neutralization of non-RBD Q677H-bearing variants and define its synergistic effects in the context of key RBD mutations.

### Q677H enhances resistance to neutralizing antibodies.

To examine neutralizing activity against SARS-CoV-2 variants, we utilized our previously reported intron-*Gaussia* luciferase-bearing lentiviral pseudotype-based neutralization assay (20) (see Text S1 in the supplemental material). We first determined the neutralizing activity of sera from 9 intensive care unit (ICU) COVID-19 patients and 9 hospitalized non-ICU patients against SARS-CoV-2 USA-WA1/2020 (wild type [WT]), D614G, and a panel of variants harboring the Q677H mutation (Fig. 1a to c). Sera were collected from both groups at least 14 days after symptom onset. For all neutralization assays, pseudotyped viruses were adjusted to comparable infectivity prior to neutralization—to prevent variations in infectivity from affecting virus neutralization. Notably, the single D614G mutant showed an increase in 50% neutralization titer (NT_50_) compared to WT (Fig. 1b and c), likely because the D614G mutation stabilizes the “open” (RBD-exposed) spike conformation (21). The Bluebird and B.1.525 variants exhibited ∼2.2-fold (*P* < 0.01)- and ∼3.6-fold (*P* < 0.01)-reduced NT_50_, respectively, compared with D614G for ICU patient samples (Fig. 1b), while non-ICU samples showed ∼2.6-fold (*P* < 0.01)-reduced NT_50_ for B.1.525 with only ∼10% reduction for Bluebird (Fig. 1c), likely due to the ∼8.7-fold-lower titer of the non-ICU samples or variations in disease state at time of serum collection.

10.1128/mBio.02510-21.1 TEXT S1 Supplemental materials and methods and supplemental references. Download Text S1, DOCX file, 0.03 MB. Copyright © 2021 Zeng et al. 2021 Zeng et al. https://creativecommons.org/licenses/by/4.0/ This content is distributed under the terms of the Creative Commons Attribution 4.0 International license.

In examining the infectivity of the lentiviral pseudotypes (see Text S1 in the supplemental material), all D614G-containing variants showed enhanced infectivity, whereas E484K exhibited decreased infectivity (29%, *P* < 0.01) and B.1.525 showed an increase (41%, *P* < 0.01) compared to D614G (Fig. 1d). Interestingly, although the Q677H mutation exhibited no drastic effect on SARS-CoV-2 S cleavage (Fig. S1a and b), it appeared to increase the spike-induced syncytium formation (Fig. S1c and d), neutralization resistance (Fig. S1e), and infectivity (Fig. S1f).

10.1128/mBio.02510-21.2 FIG S1 Impact of the Q677H mutation on SARS-CoV-2 cleavage, syncytium formation, infectivity, and neutralization in D614G and WT backgrounds. (a and b) Lysate and pseudotyped virus were collected from HEK293T cells transfected with C9-tagged SARS-CoV-2 spike and HIV-1-NL4-3-inGluc constructs. Lysate and purified virus were probed for S1 (using T62), S2 (using C9), and HIV-1 p24 (anti-p24). (c and d) HEK293T-ACE2 cells were transfected with SARS-CoV-2 spike constructs and green fluorescent protein (GFP), and syncytium formation was imaged 24 h after transfection. Images were taken under a Leica DMi8 fluorescence microscope, and the size of giant cells was quantified by using Leica LAS X software. (e) Virus neutralization assay was performed for WT spike and spike containing the Q677H mutation in the WT background. Bars indicate means with standard error, and statistical significance was determined by paired, one-tailed *t* test assuming equal variance. (f) Infectivity of the lentiviral pseudotypes bearing WT spike or spike containing the Q677H mutation in the WT background on HEK293T-ACE2 cells. Relative infectivity was calculated by setting the value of WT to 100. Download FIG S1, PDF file, 1.1 MB. Copyright © 2021 Zeng et al. 2021 Zeng et al. https://creativecommons.org/licenses/by/4.0/ This content is distributed under the terms of the Creative Commons Attribution 4.0 International license.

We next tested the neutralization capacities of sera from 40 age-matched recipients of the Moderna mRNA-1273 (*n* = 20; mean age, 35.4 years) and Pfizer BNT162b2 (*n* = 20; mean age, 35.3 years) vaccines collected 3 to 4 weeks post-second dose (see Text S1 in the supplemental material). Again, all variants, including those containing Q677H, showed decreased neutralization compared with D614G. In particular, B.1.525 and E484K/Q677H/D614G exhibited 36.5% (*P* < 0.001) and 46.5% (*P* < 0.001), respectively, decreased NT_50_ relative to D614G (Fig. 1e). Of note, E484K, N501Y, and Q677H mutants conferred 38.3% (*P* < 0.001), 17.8%, and 11.1% decreased NT_50_, respectively, compared with D614G (Fig. 1e and f).

### Q677H enhances infectivity and neutralizing antibody resistance of prevalent VOCs.

**FIG 2:**
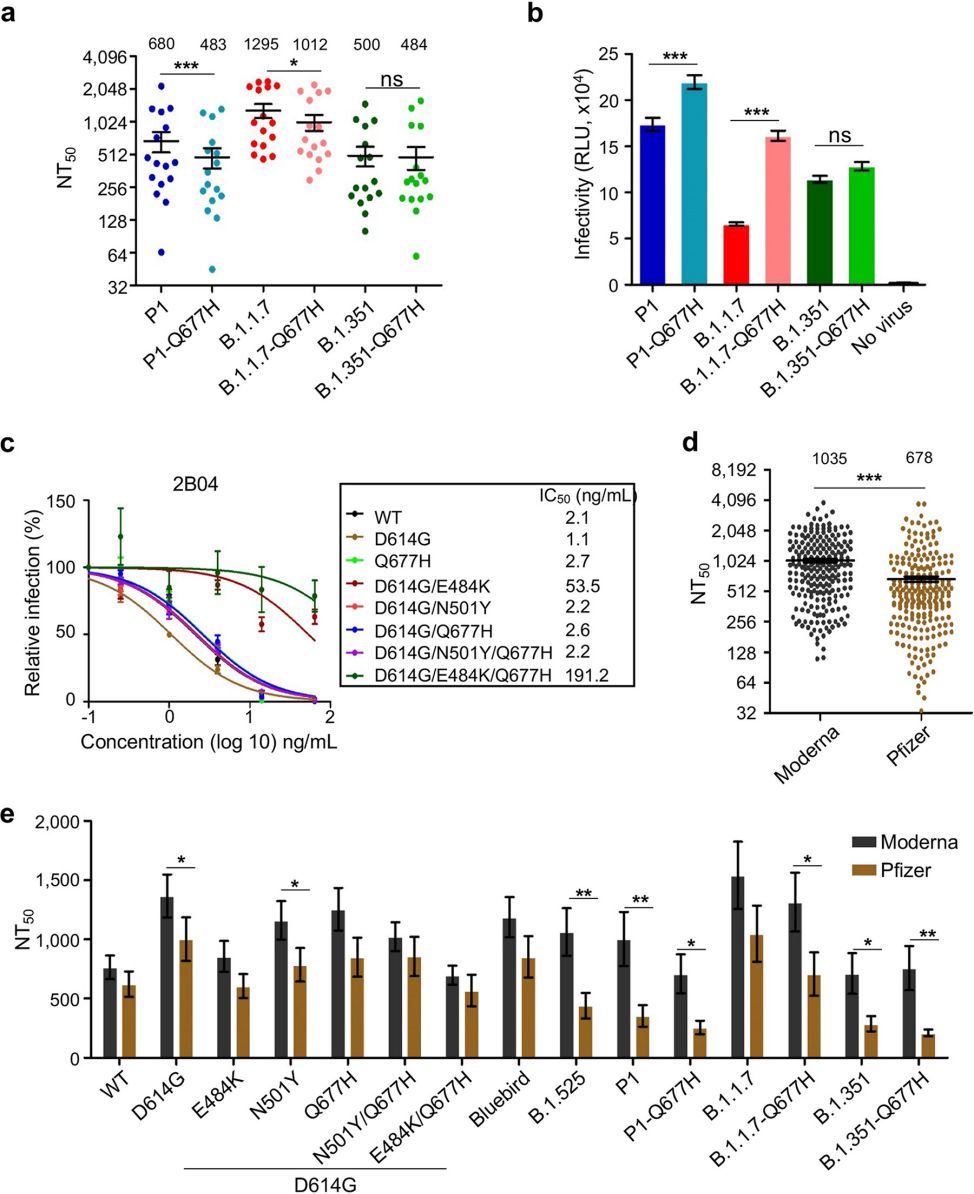
Comparison of the neutralization of VOCs B.1.1.7, B.1.351, and P1 with their counterparts containing Q677H, as well as comparison of the Moderna and Pfizer neutralizing antibody responses against Q677H-bearing variants. (a) NT_50_ values (indicated on the top) for mRNA vaccinee serum from 8 Moderna and 8 Pfizer samples, determined as in Fig. 1, against P1-501Y-V3 (Brazil), B.1.1.7 (United Kingdom), and B.1.351 (South Africa) variants with or without the introduction of the Q677H mutation. (b) Infectivity of indicated VOCs with or without Q677H. (c) Neutralization curves for monoclonal 2B04 are displayed with half-maximal inhibitory concentration (IC_50_) values, with error bars representing standard error. (d and e) The NT_50_ values for Moderna and Pfizer samples were compared against all variants tested with statistical significance being determined by unpaired, one-tailed *t* test assuming equal variance (d) and for each variant tested with statistical significance being determined by multiple unpaired *t* test (e). Bars represent means with standard error, and mean NT_50_ are displayed at the top of the plots; *, *P* < 0.05; **, *P* < 0.01; ***, *P* < 0.001; ns, not significant.

To examine the impact of the Q677H mutation emerging in major VOCs, we examined its possible role in the backgrounds of the B.1.1.7, B.1.351, and P1 variants. We found that introduction of the Q677H mutation to the B.1.1.7 and P1 spike proteins further reduced the NT_50_ of sera from mRNA vaccinees (8 Moderna and 8 Pfizer) by ∼21.9% (*P* < 0.05) and ∼29.0% (*P* < 0.001), respectively (Fig. 2a). Additionally, the Q677H mutation increased the pseudotyping viral infectivity of B.1.1.7 by 2.5-fold (*P* < 0.001) and of P1 by 26.3% (*P* < 0.001) (Fig. 2b). Interestingly, no effect of Q677H on infectivity or neutralizing antibody resistance was observed for B.1.351 (Fig. 2a and b).

Given the impact and epistasis of the non-RBD mutation Q677H on infectivity and neutralization escape, we hypothesized that it may induce conformational changes in the spike protein. To test this, we performed virus neutralization in the presence of an RBD-binding monoclonal antibody, 2B04 (22), which is known to bind the receptor binding motif of the RBD and serves as a conformation-dependent antibody. 2B04 exhibited an ∼50% reduction in neutralization of Q677H relative to WT and of Q677H/D614G relative to D614G (Fig. 2c), potentially indicating an alteration to RBD conformation. Unsurprisingly, spike proteins bearing the E484K mutation in the epitope of 2B04 (22) were not neutralized by 2B04 (Fig. 2c).

### Moderna versus Pfizer neutralization of Q677H-containing variants.

We compared the effects of age-matched Moderna and Pfizer mRNA vaccines on neutralization of all variants used in this study, including VOCs (Fig. 2d and e). Overall, the Moderna vaccine induced an ∼52.7% higher NT_50_ than did the Pfizer vaccine (Fig. 2d; *P* < 0.001). In fact, the Moderna vaccine outperformed the Pfizer vaccine against each of the viruses tested in this study, in particular B.1.525, B.1.351, and P1 (*P* < 0.01) (Fig. 2e).

### Discussion.

Although the non-RBD mutation Q677H alone in SARS-CoV-2 spike led to only modest neutralization resistance, it increased viral infectivity and syncytium formation and, importantly, had an epistatic effect when paired with certain emerging RBD mutations present in VOCs. Q677 is situated in a disordered region near the critical RRAR furin-cleavage site of SARS-CoV-2 spike (16). Thus, it is possible that Q677H might alter protease processing or spike conformation, as suggested by the increased syncytium formation and reduced neutralization by 2B04 of the D614G/Q677H spike compared to D614G; however, no dramatic effect of Q677H on furin cleavage was observed. It is possible that the effect of Q677H is masked by the presence of other mutations, including D614G. Future structural studies are required to determine the exact mechanisms by which Q677H impacts infectivity and spike conformation.

In this study, we found that the E484K mutation had a greater impact on neutralization by convalescent-phase (∼3.8-fold decrease) compared with vaccinee (∼2-fold decrease) sera, consistent with recent reports (14). Moreover, we found that Q677H increased the infectivity and neutralizing antibody resistance of B.1.1.7 and P1 spike. These conclusions are further strengthened by the neutralization profiles of K484E and H677Q reversion mutants made in the backbone of B.1.525 and Bluebird variants, where a modest effect was observed for Q677H mutants compared to K484E (Fig. S2). These findings are critical as SARS-CoV-2 VOCs continue to evolve to increase their transmissibility and resistance to vaccinee sera, including the Delta variants (2, 23–25). Interestingly, we did not find increased infectivity and neutralizing antibody resistance for Q677H in the context of the B.1.351 variant, which could be due to its preexisting strong resistance to neutralization (23) or due to the presence of other compensatory mutations. Overall, our findings underscore the need to better understand epistatic interactions between RBD and non-RBD mutations in the spike as SARS-CoV-2 evolves in the face of new immunologic challenges.

10.1128/mBio.02510-21.3 FIG S2 Comparison of the neutralization of B.1.525 and Bluebird spike-pseudotyped lentivirus with H677Q reversion mutants by vaccinated sera. NT_50_ values for vaccinee sera from 8 Pfizer (a) and 8 Moderna (b) samples, same as Fig. 2a, against B.1.525 and Bluebird variants with or without H677Q and K484E mutations. Download FIG S2, PDF file, 0.2 MB. Copyright © 2021 Zeng et al. 2021 Zeng et al. https://creativecommons.org/licenses/by/4.0/ This content is distributed under the terms of the Creative Commons Attribution 4.0 International license.
