# Erratum for Zeng et al., “Neutralization of SARS-CoV-2 Variants of Concern Harboring Q677H”

**DOI:** 10.1128/mBio.02989-21

**Published:** 2021-11-02

**Authors:** Cong Zeng, John P. Evans, Julia N. Faraone, Panke Qu, Yi-Min Zheng, Linda Saif, Eugene M. Oltz, Gerard Lozanski, Richard J. Gumina, Shan-Lu Liu

**Affiliations:** a Center for Retrovirus Research, The Ohio State University, Columbus, Ohio, USA; b Department of Veterinary Biosciences, The Ohio State University, Columbus, Ohio, USA; c Molecular, Cellular and Developmental Biology Program, The Ohio State University, Columbus, Ohio, USA; d Center for Food Animal Health, Animal Sciences Department, OARDC, College of Food, Agricultural and Environmental Sciences and Veterinary Preventive Medicine Department, College of Veterinary Medicine, The Ohio State University, Wooster, Ohio, USA; e Department of Microbial Infection and Immunity, The Ohio State University, Columbus, Ohio, USA; f Department of Pathology, The Ohio State University, Columbus, Ohio, USA; g Division of Cardiovascular Medicine, Department of Internal Medicine, Davis Heart and Lung Research Institute, The Ohio State University, Columbus, Ohio, USA; h Viruses and Emerging Pathogens Program, Infectious Diseases Institute, The Ohio State University, Columbus, Ohio, USA

## ERRATUM

Volume 12, no. 5, e02510-21, 2021, https://doi.org/10.1128/mBio.02510-21. Eugene M. Oltz’s name was incorrect in the original article. The byline should appear as shown above. The byline has been corrected online.

