# Supplementary material for: Neutralization of SARS-CoV-2 Variants of Concern Harboring Q677H
Source: mBio. 2021 Oct 5;12(5):e02510-21. doi: 10.1128/mBio.02510-21 (PMC8527387; doi:10.1128/mBio.02510-21)
Supplement: TEXT S1 [file mbio.02510-21-s0001.docx]

**TEXT S1**

**Neutralization of SARS-CoV-2 Variants of Concern Harboring Q677H**

Cong Zeng^a,b^, John P. Evans^a,b,c^, Julia N. Faraone^a,b,c^, Panke Qu^a,b^, Yi-Min Zheng^a,b^,

Linda Saif^d,h^, Eugene Otlz^e^, Gerard Lozanski^f^, Richard J. Gumina^g^, and

Shan-Lu Liu^a,b,e,h#^

^a^ Center for Retrovirus Research, The Ohio State University, Columbus, OH 43210, USA

^b^ Department of Veterinary Biosciences, The Ohio State University, Columbus,

OH 43210, USA

^c^ Molecular, Cellular and Developmental Biology Program,

The Ohio State University, Columbus, OH, USA

^d^ Center for Food Animal Health, Animal Sciences Department, OARDC, College of Food,

Agricultural and Environmental Sciences and Veterinary Preventive Medicine Department,

College of Veterinary Medicine, The Ohio State University, Wooster, OH, USA

^e^ Department of Microbial Infection and Immunity, The Ohio State University,

Columbus, OH, USA

^f^ Department of Pathology, The Ohio State University, Columbus, OH, USA.

^g^ Division of Cardiovascular Medicine, Department of Medicine,

Davis Heart and Lung Research Institute,

The Ohio State University, Columbus, OH, USA

^h^ Viruses and Emerging Pathogens Program, Infectious Diseases Institute,

The Ohio State University, Columbus, OH, USA

**Running Head:** Neutralization of SARS-CoV-2 Variants Harboring Q677H

Cong Zeng and John Evans contributed equally to this work. Order was determined based on seniority.

^#^ To whom correspondence should be addressed: Dr. Shan-Lu Liu

Tel: (614) 292-8690; Email: [liu.6244@osu.edu](mailto:liu.6244@osu.edu)

**Supplementary materials and methods**

**Patients and Vaccinated Individuals.** De-identified vaccinated individual serum samples were collected under approved an approved IRB protocol (2020H0228). Serum was collected 3-4 weeks post-vaccination with the second dose of Pfizer (n=20) or Moderna (n=20) SARS-CoV-2 mRNA vaccine. The vaccinated groups were roughly age-matched, with Pfizer vaccinated individuals having a mean age of 35.3 years, ranging from 22 years to 46 years. Moderna vaccinated individuals had a mean age of 35.4 years, ranging from 25 years to 56 years.

ICU patient samples were collected as previously described (1) under an approved IRB protocol (OSU 2020H0228). Briefly, serum was isolated using Gold top serum-separator tubes (Beckton Dickinson) or Red top clotting tubes (Beckton Dickinson). Serum was allowed to separate for 24 hrs at room temperature. Severely hemolytic samples were rejected.

**Cell Lines and Maintenance.** HEK293T cells (ATCC CRL-11268, CVCL_1926) and HEK293T-ACE2 cells (BEI NR-52511) were maintained in DMEM (Gibco, 11965-092) supplemented with 10% FBS (Sigma, F1051) and 1% penicillin/streptomycin (HyClone, SV30010). Both cell lines were maintained at 37°C, and 5% CO_2_ in tissue culture treated, sterile 10cm dishes (FisherScientific, FB012924).

**Constructs.** The construct used for the production of lentiviral pseudotypes was HIV1–NL4.3–inGluc (1-3), which was originally obtained from David Derse’s lab at NIH (National Cancer Institute, Frederick, Maryland, USA) and Marc Johnson’s lab at the University of Missouri (Columbia, Missouri, USA). This construct is based on a ΔEnv pNL4.3 HIV-1 vector and contains an anti-sense Gaussia luciferase (Gluc) gene with a sense intron. Gluc is secreted in mammalian cell culture (4), and the intron and anti-sense orientation of the Gluc gene prevents the production of Gluc in the virus producer cells (1-3). The codon-optimized B.1.1.7, P1-501Y-V3, B.1.351, B.1.525, and Bluebird SARS-CoV-2 S constructs were synthesized by GenScript and subsequently cloned into a pcDNA3.1 vector by restriction enzyme cloning with Kpn I and BamH I. The K484E and H677Q reversion mutants were made in the context of B.1.525 and Bluebird variants by PCR-based site-directed mutagenesis. The wild-type S construct, pcDNA3.1-SARS-CoV-2-S-C9 which encodes codon-optimized full-length spikes tagged with C9 at the C-terminus, and was a gift from Fang Li’s lab at the University of Minnesota (St. Paul, Minnesota, USA). All other mutants were produced from these templates via site-directed mutagenesis (5). Successful mutagenesis was confirmed by Sanger sequencing by the Genomics Shared Resources at the OSU Comprehensive Cancer Center.

**Antibodies.** Primary antibodies used for western blotting were anti-coronavirus spike (Sino Biological, #40150-T62) for detection of S1, anti-rhodopsin (1D4) (Santa Cruz Bio, sc-57432) for detection of S2, anti-p24 (Abcam, ab63917) for detection of HIV Gag, and anti-GAPDH (Santa Cruz Bio, sc-25778) for detection of cellular GAPDH. Secondary antibodies used for western blotting included anti-Mouse IgG-Peroxidase (Sigma, #A5278) and anti-Rabbit IgG-Peroxidase (Sigma, #A9169).

**Virus Production.** Lentiviral pseudotyped virus was produced as previously described (1). Briefly, HEK293T cells were transfected with HIV-1-NL4.3-inGluc and the various S protein constructs in a 2:1 ratio using polyelthylenimine (PEI). The resulting cell supernatants were harvested 24 hr, 48 hr, and 72 hr post-transfection and were pooled, aliquoted, and stored at -80°C. Aliquots of S variant viruses were thawed and used to infect HEK293T-ACE2 cells for 6 hrs, and then media was changed. Gaussia luciferase activity was measured 48 hrs after infection to determine the relative infectivity of the produced virus. Virus was adjusted by volume to comparable infectivity prior to performing virus neutralization assays.

**Pseudotype Virus Neutralization Assays.** Pseudotyped virus neutralization assays (VNAs) were performed as previously described (1). In a flat-bottom 96-well plate (Cellstar, 655180), patient/vaccinated individual serum was 4-fold serial diluted, resulting in a final volume of 60 μL. Subsequently, 100 μL of pseudotyped virus was added to the samples resulting in a final set of dilutions of 1:40, 1:160, 1:640, 1:2560, 1:10240, and no serum. Virus was allowed to neutralize for 1 hr at 37°C and 5% CO_2_. Neutralized virus was subsequently added to HEK293T-ACE2 cells seeded at 2 x 10^4^ cells/well. The infection was allowed to proceed for 6 hrs, after which the media on the cells was removed and replaced with 100 μL fresh media. At 48 hrs and 72 hrs after infection, 20 μL of media was collected from the cells and transferred to a white, flat-bottomed, polystyrene 96-well plate (Thermo Scientific, 236108). 20 μL of Gaussia luciferase substrate (0.1 M Tris (MilliporeSigma, #T6066) pH 7.4, 0.3 M sodium ascorbate (Spectrum, S1349), 10 μM coelenterazine (GoldBio, CZ2.5)) was added to the media and immediately read by a BioTek Cytation5 plate-reader.

**Purification of pseudoviral particles and western blotting.** Supernatant containing pseudotyped virus was collected and purified by ultracentrifugation through 20% sucrose cushion. Western blotting of purified viral particles and cell lysates was performed as previously described (3). In brief, transfected cells were collected and washed with PBS, lysed in RIPA buffer (50mM Tris, pH 7.5, 150 mM NaCl, 1mM EDTA, 1% NP40, 0.1% SDS and protease inhibitor cocktail) for 40 min on ice, followed by centrifugation for 10 min, 12,000 x g at 4 ℃. Cell lysates were then boiled at 100 ℃ for 10 min with 1xSDS loading buffer. Purified viral particles were dissolved in 1xSDS loading buffer and boiled at 100 ℃ for 10 min. Samples were run on 10% SDS-PAGE gels, transferred to PVDF membranes, and probed with primary and secondary antibodies, analyzed by Amersham Imager 600 (Thermofisher).

**Syncytia formation.** HEK293T-ACE2 cells were seeded in 24-well plate transfected with 300 ng SARS-CoV-2 Spike WT or mutant constructs along with 100 ng GFP, syncytia formation was imaged 24hrs after transfection (Leica DMi8). The extent of cell-cell fusion was compared by measuring size of giant cells using software “Leica LAS X”.

**Quantification and Statistical Analysis.** NT_50_ values were calculated from VNA output using a non-linear regression with least-squares fit in GraphPad Prism5 for Windows, GraphPad Software, San Diego, California USA, www.graphpad.com. Statistical significance between multiple groups was similarly calculated using one-way repeated measures ANOVA with Bonferroni post-tests in GraphPad Prism5 with a p-value of 0.05 being considered significant. Statistical significance between two groups was calculated using an unpaired, one-tailed t-test assuming equal variance also in GraphPad Prism5 with a p-value of 0.05 being considered significant.

**Supplementary references:**

1. Zeng C, Evans JP, Pearson R, Qu P, Zheng Y-M, Robinson RT, Hall-Stoodley L, Yount J, Pannu S, Mallampalli RK. 2020. Neutralizing antibody against SARS-CoV-2 spike in COVID-19 patients, health care workers, and convalescent plasma donors. JCI insight 5.

2. Mazurov D, Ilinskaya A, Heidecker G, Lloyd P, Derse D. 2010. Quantitative comparison of HTLV-1 and HIV-1 cell-to-cell infection with new replication dependent vectors. PLoS Pathog 6:e1000788.

3. Yu J, Li M, Wilkins J, Ding S, Swartz TH, Esposito AM, Zheng Y-M, Freed EO, Liang C, Chen BK. 2015. IFITM proteins restrict HIV-1 infection by antagonizing the envelope glycoprotein. Cell reports 13:145-156.

4. Goerke AR, Loening AM, Gambhir SS, Swartz JR. 2008. Cell-free metabolic engineering promotes high-level production of bioactive Gaussia princeps luciferase. Metabolic engineering 10:187-200.

5. Liu H, Naismith JH. 2008. An efficient one-step site-directed deletion, insertion, single and multiple-site plasmid mutagenesis protocol. BMC biotechnology 8:1-10.
