# Supplementary material for: Neutralization of SARS-CoV-2 Variants of Concern Harboring Q677H
Source: mBio. 2021 Oct 5;12(5):e02510-21. doi: 10.1128/mBio.02510-21 (PMC8527387; doi:10.1128/mBio.02510-21)
Supplement: FIG S1 [file mbio.02510-21-sf001.pdf]

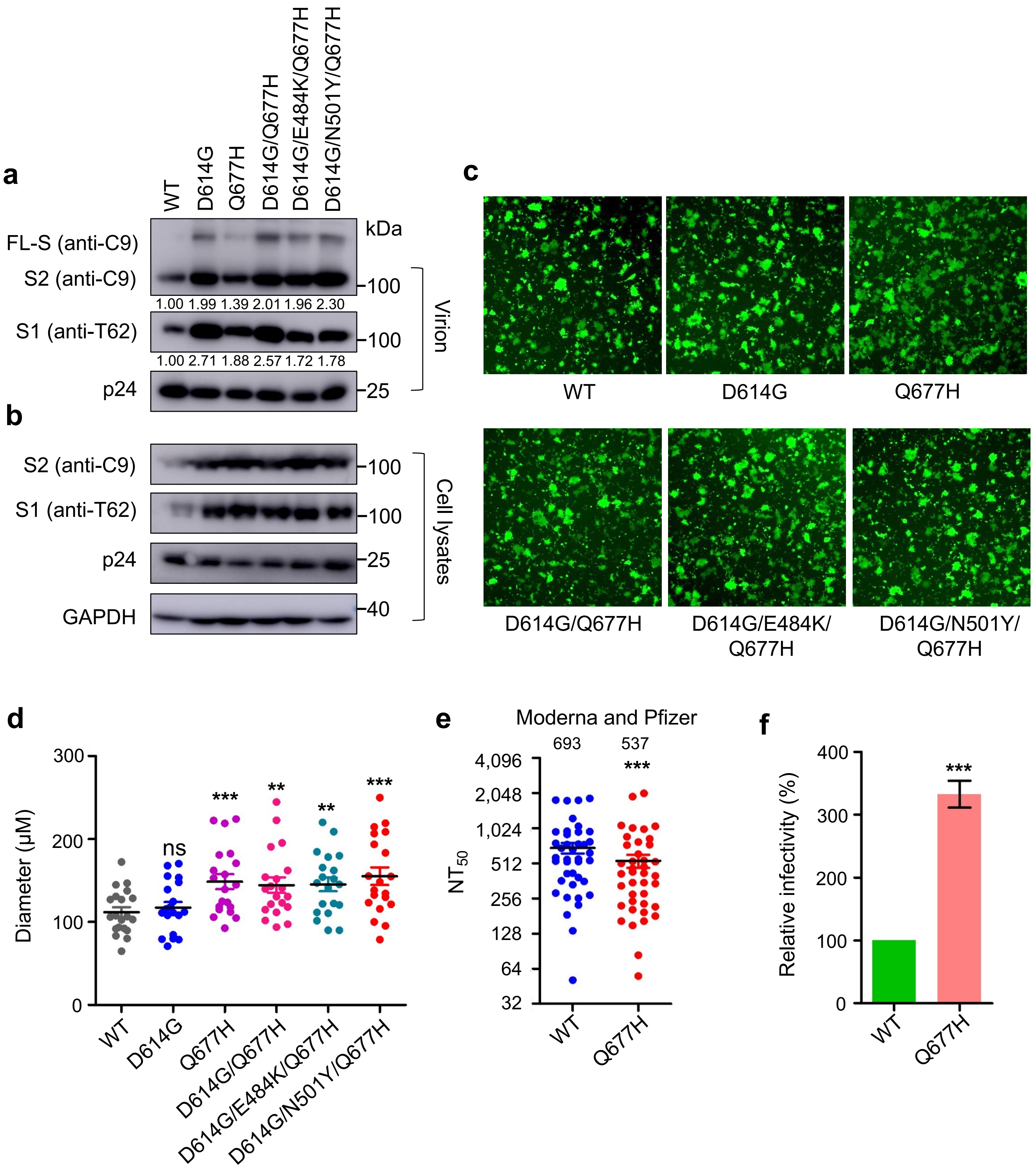

Figure S1. Impact of the Q677H mutation on SARS-CoV-2 cleavage, syncytia formation, infectivity and neutralization in D614G and WT backgrounds. (**a**) and (**b**) Lysate and pseudotyped virus were collected from HEK293T cells transfected with C9-tagged SARS-CoV-2 Spike and HIV-1-NL4-3-inGluc constructs. Lysate and purified virus were probed for S1 (using T62), S2 (using C9), and HIV-1 p24 (anti-p24). (**c**) and (d) HEK293T-ACE2 cells were transfected with SARS-CoV-2 Spike constructs and GFP and syncytia formation was imaged 24 hrs after transfection. Images were taken under fluorescence microscope Leica DMi8 and the size of giant cell was quantified by using software "Leica LAS X". (**e**) Virus neutralization assay was performed for WT Spike and Spike containing the Q677H mutation in the WT background. Bars indicate means with standard error, and statistical significance was determined by paired, one-tailed t-test assuming equal variance. (**f**) Infectivity of the lentiviral pesudotypes bearing WT Spike or Spike containing the Q677H mutation in the WT background on HEK293T-ACE2 cells. Relative infectivity was calculated by setting the value of WT to 100.
