# Supplementary material for: Neutralization of SARS-CoV-2 Variants of Concern Harboring Q677H
Source: mBio. 2021 Oct 5;12(5):e02510-21. doi: 10.1128/mBio.02510-21 (PMC8527387; doi:10.1128/mBio.02510-21)
Supplement: FIG S2 [file mbio.02510-21-sf002.pdf]

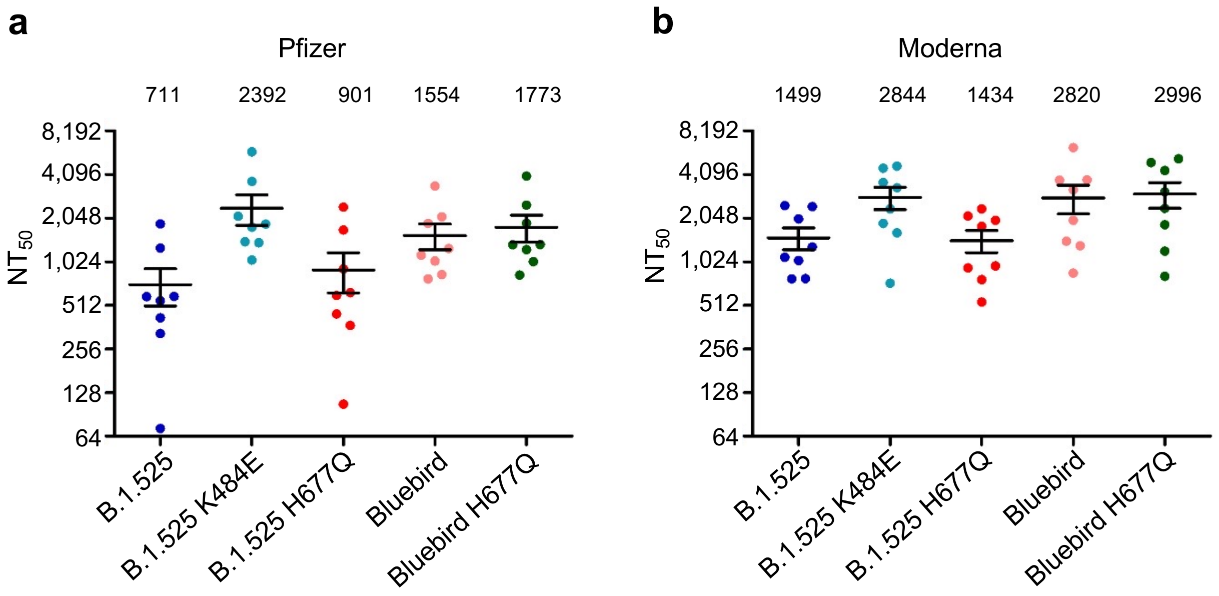

Figure S2. Comparison of the neutralization of B.1.525 and Bluebird spike-pseudotyped lentivirus with H677Q reversion mutants by vaccinated sera. NT_{50} values for vaccinee sera from 8 Pfizer (**a**) and 8 Moderna (**b**) samples, same as figure 2a, against B.1.525 and Bluebird variants with or without H677Q and K484E mutation.
